# Sensing of Abiotic Stress and Ionic Stress Responses in Plants

**DOI:** 10.3390/ijms19113298

**Published:** 2018-10-24

**Authors:** Yu Zhang, Yang Lv, Noushin Jahan, Guang Chen, Deyong Ren, Longbiao Guo

**Affiliations:** State Key Lab for Rice Biology, China National Rice Research Institute, Hangzhou 310006, China; zhangyu881005@163.com (Y.Z.); yanglv_simon@foxmail.com (Y.L.); nowshin012@gmail.com (N.J.); chenguang0066@126.com (G.C.); rendeyongsd@163.com (D.R.)

**Keywords:** abiotic stress, ionic stress, sensing, response, CBL−CIPK

## Abstract

Plants need to cope with complex environments throughout their life cycle. Abiotic stresses, including drought, cold, salt and heat, can cause a reduction in plant growth and loss of crop yield. Plants sensing stress signals and adapting to adverse environments are fundamental biological problems. We review the stress sensors in stress sensing and the responses, and then discuss ionic stress signaling and the responses. During ionic stress, the calcineurin B-like proteins (CBL) and CBL-interacting protein kinases (CBL−CIPK) complex is identified as a primary element of the calcium sensor for perceiving environmental signals. The CBL−CIPK system shows specificity and variety in its response to different stresses. Obtaining a deeper understanding of stress signaling and the responses will mitigate or solve crop yield crises in extreme environments with fast-growing populations.

## 1. Introduction

Plants cannot escape adverse environmental conditions [1]. Due to the constantly changing environment, abiotic stress is the main factor affecting crop growth and productivity, as plants are sessile organisms. Abiotic stress includes soil salinity, drought and extreme temperature conditions (cold or hot) [2]. Anthropogenic activities directly affect global climate change. Identifying the mechanisms through which plants counteract abiotic stress and maintain their growth and survival holds significance for plants in coping with global climate change [3,4,5].

There are inherent physical, morphological and molecular limitations to the plant’s ability to respond to different abiotic stresses [6]. Although plants differ physically, morphologically and molecularly between wild-type and modern cultivars in terms of their stress sensing ability, their stress sensing mechanisms are still poorly understood. To overcome this limitation, an initial step in decoding the process involves stress sensor signals that are produced by the plant cell. Studies on calcineurin B-like proteins (CBLs) and their targets, CBL-interacting protein kinases (CIPKs), generally known as the CBL−CIPK network, provide useful models for understanding the mechanisms of stress signal transduction in plants [2].

Cold stress can rigidify the plasma membrane and rearrange the cytoskeleton so calcium can pass through calcium channels into the cytoplasm [7]. The calcium signal is then decoded by a calcium-binding protein, which leads to downstream actions that deliver the cold signal [7]. *COLD1*, a quantitative trait locus gene identified in rice that encodes a regulator of G-protein signaling localized on the plasma membrane and endoplasmic reticulum, was found to cause the G-protein α subunit to initiate the guanosine triphosphatase (GTPase) activity of regulator of G-protein activate 1 (RGA1) [8]. COLD1/RGA1 represents a potential calcium permeable channel, as a lack of COLD1 affects calcium (Ca^2+^) signaling with cold stress in rice [7,8]. Phytochromes (Phy), plant photoreceptors, regulate photomorphogenesis, and PhyB, the primary photoreceptor, controls plant growth in *Arabidopsis*, both in cold and high temperature (HT) stress [7,9,10]. Under normal and HT conditions, phytochrome interacting factor 4 (PIF4), a basic helix-loop-helix transcription factor, forms part of the central regulatory hub mediating the diurnal growth of plants [9]. It was proposed that the phyB−PIF4 signaling module plays an important role in balancing plant growth and defenses during the response to HT stress [11]. PIF4 can integrate brassinosteroid (BR) and gibberellin (GA) signaling. Evidence has shown that PIF4 directly binds to the promoters of *DWARF4* (*DWF4*) and *BRASSINOSTEROID-6-OXIDASE2* (*BR6ox2*), and then promotes the expression of these BR biosynthesis genes [12].

Soil salinization is a worldwide problem that seriously threatens agriculture as it restricts the growth and yield of crops [13]. Salt stress includes two types: osmotic stress and ionic stress. Rice is known to be a salt-sensitive crop and the annotations of the rice genome sequence enable the study of the functional genomics of the salt stress response [14]. *Oryza sativa Salt tolerance activation 2* (*OsSta2*) was identified and studied in rice. Under salt stress, OsSta2-Ox (overexpression) plants were more tolerant to osmotic stress and could maintain a much healthier growth pattern than wild type (WT) seedlings in response to mannitol treatment, which indicates that *OsSta2* may respond to salt and drought stress [15]. Studies have found that some RNA functions are also involved in crop salt tolerance mechanisms [16,17,18,19]. With RNA-seq and sRNA-seq, there were 2574 mRNAs and 76 miRNAs, respectively, that were differentially expressed in citrus root under salt and dehydration treatments [17]. In maize, eight novel miRNAs and their targets, in response to salinity stress, have been identified. A total of 37 potential new miRNAs were selected based on the same criteria in response to salt stress [16]. The interaction between miRNAs and their targets may also play important roles in response to salinity.

Multiple Mitogen-activated protein kinases (MAPKs) have been observed, which can respond to abiotic stimuli such as salt, drought, cold, heat and wounding, as well as to growth and developmental signals [20]. Abscisic acid (ABA) has been shown to modulate the transcription, protein accumulation and kinase activity of several MAPK signaling components [20]. The ABA-signaling pathway is central to drought and salt stress responses in plants [2,21]. The interactions of CIPK1, CBL1 and CBL9 form different complexes that seem to work in distinct processes, including drought-responsive gene expression and ABA responses.

Therefore, understanding the physiological and molecular aspects of plant stress sensing functions and improving plant stress resistance are critical for agricultural growth and productivity.

## 2. Sensing of Organellar Stress

**Figure 1:**
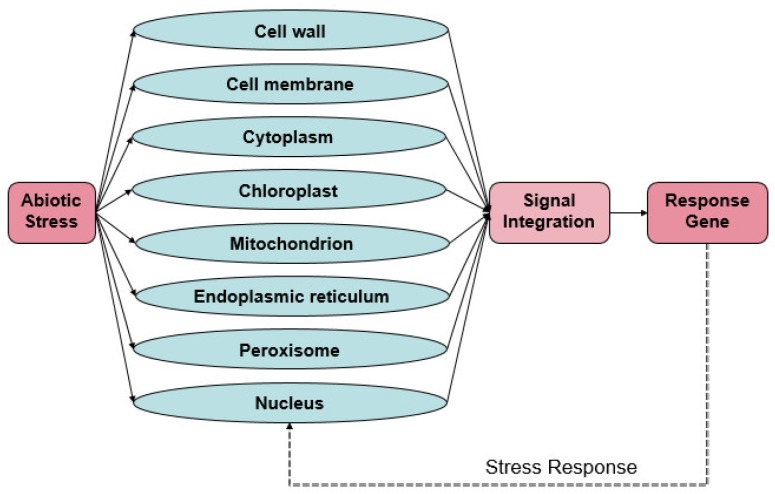
Stress sensing and signaling; a model of the response to abiotic stresses in different organelles. The signal generated by organelles can cause gene expression and cellular activities, which can restore cellular homeostasis under abiotic stress. Arrows indicate activation and signal transduction, and dashed lines indicate response.

Plant cells are capable of sensing various environmental signals in response to different abiotic stress conditions. The plant then demonstrates variations in gene expression, metabolism and physiology [2]. Research has revealed abiotic stress signaling perception in plants. Sensors that recognize the initial stress signal initiate cascades that transmit the signal to different organelles and activate transcription factors (TFs) that induce the expression of a particular set of genes. A single sensor can regulate the branches of the signal cascade, which is triggered by one aspect of the stress condition (Figure 1).

Stress-sensing frequently occurs on the cell surface or at the cell membrane [22]. From there, the signal can be relayed to different organelles. Protein folding in the endoplasmic reticulum (ER), referred to as ER stress, is widely recognized as an important cellular response to stress, which can be caused by both biotic and abiotic stress [2,23]. ER membrane-associated transcription factors, basic leucine zipper (bZIP) proteins, are important for restoring ER homeostasis [24]. The bZIP transcription factors (TFs) constitute one of the largest families in plants with nearly 15,498 genes from 166 species [25].

Based on the plant transcription factor database, different kinds and numbers of TFs are found in different plant species, which are identified as loci and classified into different families (Table 1). For instance, the gene annotation from TAIR10 is used to identify transcription factors (TFs) of *Arabidopsis thaliana*. According to the family assignment rules, 2296 TFs (1717 loci) have been identified and classified into 58 families [25].

The production of reactive oxygen species (ROS), including superoxide anion, hydrogen peroxide (H_2_O_2_), hydroxyl radicals and singlet oxygen, mainly affects chloroplasts [26,27]. As the major cellular component for photosynthesis, chloroplasts are highly exposed to ROS damage since photosynthesis is a source of ROS, especially H_2_O_2_ [28]. Like chloroplasts, mitochondria and peroxisomes can generate retrograde signals. They produce ROS and many metabolites that may affect calcium signaling, some of which may serve as retrograde signals [29,30,31].

The primary cell wall of the plant is composed of cellulose fibrils, which are connected by hemicellulose tethers and embedded in a pectin gel [22]. The pectins are often modified when exposed to drought stress. By comparing the cell wall changes of two different drought-resistant wheat varieties under stress, an increase in the pectin polymers rhamnogalacturonan I and II (RGI and RGII) side chains was found to probably be due to the hydrogel formation of pectin, which limited the damage to the cells [32]. The cell wall also contains phenolics, enzymes, proteins, and Ca^2+^. Salt, drought, and other osmotic stress treatments can lead to the accumulation of ROS in the cell wall [22]. The dirigent (DIR) proteins in plants modulate the cell wall metabolism during abiotic and biotic stress [33]. Some proteins in the cellulose synthase complex help connect the complex to microtubules for plant growth during salt stress [34]. Members of different receptor-like kinases perceive the stress, which includes a large family of integral plasma membrane proteins. These kinases, upon perceiving stress, can transmit their signal into the cell [35]. Constitutive expression of receptor-like protein kinase 1 (RPK1) can cause upregulation of a number of stress-induced genes and enhance abiotic stress tolerance in *Arabidopsis* [36].

As well, the changes in organellar gene expression (OGE) in response to developmental and environmental changes, and perturbations of OGE homeostasis, regularly result in the activation of tolerance responses [37].

## 3. Ionic Stress Signaling

High salinity stress causes an ion homeostasis imbalance inside the plant cell. Plant cells produce an ionic stress signal. The salinity stress signal is then perceived by a receptor or salt sensor present at the plasma membrane of the cell, which is generally regulated by the coordinated action of various salt overly sensitive (SOS) pathways and ion pumps, and its downstream interacting partners, which ultimately results in the efflux of excess ions

### 3.1. Sodium

**Figure 2:**
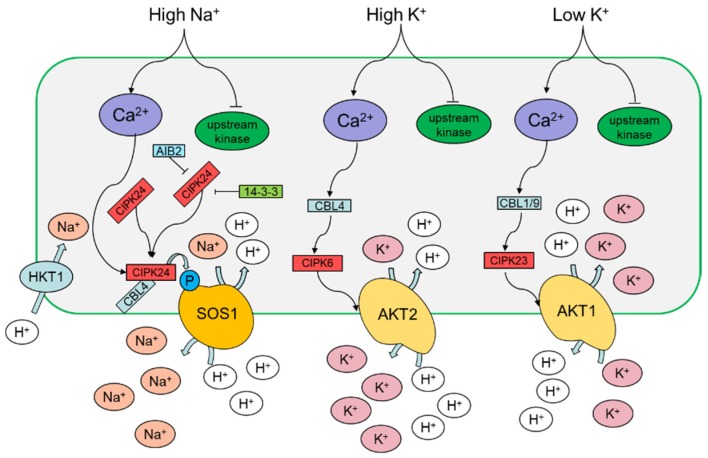
The abiotic stress of high sodium, low potassium and high potassium stress. Different colors indicate different pathways. AKT1: *Arabidopsis* K^+^ transporter 1, AKT2: *Arabidopsis* K^+^ transporter 2, HKT1: high-affinity potassium transporter 1, SOS1: salt overly sensitive. Arrows indicate activation, and bars indicate inhibition.

Detrimental ionic stress, especially salt stress, including chloride (Cl^−^), carbonate (CO_3_^2−^) and sulfate (SO_4_^2−^) salts of sodium (Na^+^), magnesium (Mg^2+^) or calcium (Ca^2+^), is harmful to plants and occurs in different geographical areas around the world [38]. Na^+^ has a deleterious effect when it accumulates in cells and certain tissues, directly interfering with cellular functions. Plants offset the initial osmotic components of salt stress by adjusting the osmotic gradient, although the accumulation of Na^+^ can lead to toxic effects in the long-term [39]. Na^+^ affects the hydration shell of other molecules, causes damage to the cell wall, disturbs the K^+^/Na^+^ ratio of cells by several mechanisms and impairs plant physiology [40,41,42]. Plants use a calcium-dependent protein kinase pathway, known as the SOS pathway, for salt stress signaling and Na^+^ tolerance [21]. Until recently, a sensor or receptor of Na^+^ has not been identified in plants [43,44]. In plants, the Ca^2+^ sensor relay proteins do not have any kinase activity (Figure 2); they interact with sensor responder proteins to modulate downstream reactions after binding to Ca^2+^. The name CBL has been widely used to refer to a family of EF-hand calcium-binding proteins [45].

In the plant SOS pathway, SOS3 (CBL4), an EF-hand calcium-binding protein, senses the cytosolic calcium signal. SOS3 subsequently interacts with, and activates, the CBL-interacting protein kinase SOS2 (CIPK24) [46,47]. Despite the ability of SOS3 to bind Ca^2+^, the in vitro formation and activation of SOS3-SOS2 complexes was not enhanced by increasing Ca^2+^ concentrations [48]. The activated SOS2 phosphorylates and activates SOS1, a Na^+^/H^+^ antiporter at the plasma membrane [2,21]. Dysfunction in any of the *SOS* genes increases the sensitivity of the mutant plants to salt stress. The *sos1* mutants appeared to be the most salt-sensitive, followed by *sos2* mutants exhibiting intermediate salt sensitivity, with *sos3* mutants being the least salt sensitive [49].

SOS1 and high-affinity potassium transporter 1 (HKT1) have antagonistic functions. The HKT protein family has been shown to be critical for salinity tolerance in plants [50]. HKT1 in *Arabidopsis* is a Na^+^ importer, expressed in parenchyma cells and other cells in the vascular system throughout the plant [51,52]. In the roots, HKT1 unloads Na^+^ from the xylem in order to limit the amount of Na^+^. In the leaves, HKT1 was thought to be able to load Na^+^ into the phloem and recycle it back to the root [53]. A mutation of HKT1 caused obvious sensitivity to salt stress, but inhibited the salt sensitivity of SOS mutants in the culture medium [53].

Under salt stress, *OsHKT1;1*, a Na^+^ transporter expressed in the vascular tissue of rice shoots, enhances Na^+^ ion exclusion from the cell [54,55]. Mostly in monocot plants, HKT1 was found to function in the vascular tissue of roots and shoots, and regulate Na^+^ ion movement from root to shoot. In salt-tolerant landraces, unlike *OsHKT1;1*, the *OsHKT1;4* gene more efficiently contributes to Na^+^ exclusion from leaf blades at leaf sheaths, compared with the *japonica* rice species [56]. In *japonica* rice cultivars, *OsHKT1;4* down-regulates Na^+^ ion concentration in leaves by regulating Na^+^ ion movements from stem to leaves at the vegetative stage, but has no impact on lowering the high Na^+^ ion concentration directly from leaves [57,58]. A study showed that mutants with an overexpression of *OsHKT1;4* are more sensitive to salt than WT plants [59]. *OsHKT1;5*, known as the *SKC1* gene, was determined to be the salt-tolerant determinant gene through quantitative trait loci (QTL) analysis [60]. OsHKT1;5-dependent translocation of Na^+^ in roots, leaf sheaths, and stems is a key mechanism of salt tolerance throughout the growth stages of rice and *Arabidopsis*. AtHKT1 was expressed mainly in the phloem tissues and was proposed to function in the recirculation of Na^+^. The function of OsHKT1;5 is crucial, including the protection of the next generation seeds and vital leaf blades under salt stress [61].

SOS3 has an N-terminal myristoylation signal peptide crucial for SOS3 function in salt stress. SOS3-like calcium-binding proteins are phosphorylated by interacting with SOS2-like protein kinases, and phosphorylation appears important for the activation of kinases by calcium-binding proteins [62]. 14-3-3 proteins, regulatory factors, are highly conserved in eukaryotes and function in almost every aspect of plant growth and development. 14-3-3 proteins interact with SOS2 and can repress SOS2 activity under non-stress conditions, but sodium can reduce the interaction between 14-3-3 proteins and SOS2 to activate an SOS pathway for salt tolerance [63]. 

SOS2, a large family of protein kinases, has two similar kinases in yeast and mammals: sucrose nonfermenting 1 (SNF1) and AMP-activated protein kinases (AMPKs). In plants, these proteins are generally referred to as SNF1-related kinases (SnRKs), which play a major role in regulating gene expression in plant cells [64]. In several plants, members of the SnRK subfamily have been identified [65,66]. The first identified plant SnRK is the rice homolog of SNF1-encoded protein-serine/threonine kinase (RKIN1), which was found in rye endosperm [67]. According to its amino acid sequences, and based on the evolutionary relationships, the SnRK family has been divided into three subfamilies: SnRK1, SnRK2, and SnRK3 [68]. The SnRK1 subfamily has a highly conserved (ca. 62–64% amino acid identity) N-terminal catalytic domain that has three members [68,69]. Through gain of function experiments, the SnRK1 subfamily was shown to complement yeast snf1-defective mutants. This suggested that the SnRK1 subfamily is involved in glucose signaling and transcription regulation [70]. Compared to the members of the SnRK1 subfamily, the SnRK2 subfamily has a conserved (ca. 42–46% amino acid identity) N-terminal catalytic domain, and has relatively short C-terminal conserved domains. In rice, the SnRK2 subfamily has 10 members, OsSAPK1−OsSAPK10 [71], and SnRK2 subfamily proteins that are essential to both osmotic stress responses and ABA signaling [72,73].

When the Ca^2+^ concentration changes, plants use a calcium effector protein to sense this signal, and then manage the external stimulation by regulating the expression of the plant stress gene. All these effector proteins have an EF-hand domain, defined by its helix-loop-helix secondary structure, to bind Ca^2+^ [74]. SnRK3s are rare protein kinases in plants, called calcineurin B-like calcium sensor-interacting protein kinases (CIPK or PKS), and SnRK3s interact with one or more members of the family of SOS3-like calcium-binding proteins (SCaBPs or CBLs) through a common motif known as NAF/FISL in the N-terminal regulatory region of the kinases [48,75].

### 3.2. Potassium

Potassium (K^+^) is the most commonly found cation in living plant cells. It is involved in many aspects of plant growth and development. It can affect all aspects of crop production and tolerance to various abiotic stresses [76]. Thus, the maintenance of K^+^ ion transporters and channels across the plasma membrane is essential for proper K^+^ homeostasis in plants. The large number of possible CBL−CIPK combinations suggests that the Ca^2+^−SOS3−SOS2 signaling pathway is widely used in plants. The maintenance of a high cytosolic K^+^/Na^+^ ratio is essential for plant salt tolerance. During low potassium (K^+^) stress, which presumably triggers a cytosolic calcium signal, the CBL1/CBL9−CIPK23 module activates the affinity K^+^ transporter (AKT) for K^+^ uptake (Figure 2) [77]. AKT1 is one of the most important K^+^ transporters in *Arabidopsis*. It mediates continuous growth by absorption of K^+^ by plant roots through various exogenous K^+^ concentrations and promotes high affinity K^+^ uptake in the low-K^+^ concentration range [78]. The results of *cipk23*, *cbl1*/*cbl9*, and *akt1* mutants showed similar reduced growth and chlorotic leaves under low K^+^ conditions [78,79,80]. Instead of mutant experiments, AKT1 overexpressed (OE) in *Arabidopsis* did not show any significant improvement in growth when they were grown in low K^+^ conditions, whereas At/PeCBL1, AtCBL9, and AtCIPK23 OE plants demonstrated a comparative tolerance compared to control plants under the same conditions [81,82]. In rice, the inward K^+^ channel, OsAKT1, functions in K^+^ uptake in rice roots, whose activity is regulated by OsCBL1 and OsCIPK23 [83]. The CBL−CIPK network is involved in the negative regulation of AKT1 activity. AtCBL10 is thought to be directly bound to AKT1 in competition with AtCIPK23, so AtCIPK23 stops binding and activating AKT1 [84]. OsAKT1 is expressed in almost all rice tissues and organs, which contributes considerably to K^+^ uptake in a wide range of external K^+^ concentrations. OsAKT1 impacts stomatal conductance during osmotic stress, which is important for water stress [76].

AKT2 is another K^+^ transporter involved in moving K^+^ across the plasma membrane [85,86,87]. The CBL4−CIPK6 complex controls the plasma membrane targeting of the *Arabidopsis* K^+^ channel AKT2 (Figure 2) [88]. The (de)phosphorylation network regulates the functional switch from influx to efflux. In *Arabidopsis*, protein phosphatase 2C (AtPP2CA) dephosphorylation was found to be able to repress the ability of AKT2 to move K^+^ out of the cell [89]. The knockout mutant *akt2* reduced K^+^ dependence of the phloem and affected sugar loading into the phloem [87]. The single knockout mutants *cipk6* and *cbl4* reduced growth and delayed bolting, which is similar to the *akt2* mutant [85,86,88]. Moreover, CBL2 and CBL3 may function in the regulation of K^+^ transport between the vacuole and cytoplasm [90].

The H^+^-ATPases are important components in the initial sensing in plant responses to K^+^ deficiency [91]. The plant K^+^ transporters are mainly derived from several gene families, including KUP/HAK/KT, HKT, NHX, and CHX [92]. *OsHAK1*, *OsHAK2* and *OsHAK5*, as K^+^ transporters, play important roles in K^+^ acquisition and distribution in rice [93]. The over-expression of *OsHAK1* significantly improved salinity tolerance and drought tolerance [93,94,95]. The rice OsHAK2 protein is sensitive to extracellular Na^+^ and transports Na^+^ more effectively than K^+^ [96]. The over-expression of *OsHAK5* in rice plants increased the shoot [K^+^]/[Na^+^] ratio and enhanced the salt tolerance of the plant. OsHAK5 plays an important role in the process of obtaining potassium from roots that are faced with low exogenous potassium and upward migration of potassium, transporting potassium from roots to aboveground parts in K-deficient rice plants [97].

### 3.3. Nitrate

Nitrogen is important for crop production and plant growth, and is highly regulated and coordinated with other transport and metabolic pathways [98,99]. Plants mainly uptake NO_3_^−^ as the nitrogen source [100]. There are two systems for nitrate uptake: the high-affinity system and the low-affinity system for nitrate uptake [101,102]. The high-affinity system is induced in nitrate deficit, while the low-affinity system is used for the primary nitrate uptake and response under a sufficient situation [101,102]. In plants, there are three nitrate transporter families: nitrate transporter 1 (NRT1), nitrate transporter 2 (NRT2) and chloride channel (CLC). The first identified nitrate transporter was NRT1, or chlorate resistant 1 (CHL1), which is a dose-dependent master controller of multiple signaling mechanisms, capable of responding to a wide range of soil nitrate levels [103,104]. There are 53 AtNRT1, 7 AtNRT2 and 7 AtCLC that have been identified in *Arabidopsis* [103,105,106]. Among them, AtNRT2.1 and AtNRT2.2 are engaged in high-affinity uptake. AtNRT1.1 is involved in both high- and low-affinity uptake of NO_3_^−^, whereas AtNRT1.2 works in low-affinity uptake [107,108]. In rice, up to 250-fold increases in the induction of gene expression after nitrate resupply were observed for the high-affinity transporters OsNRT2.1 and OsNRT2.2 [109].

When faced with a lack of nitrate, AtCBL19-AtCIPK23 compounds are responsible for NRT1.1 phosphorylation, which improves the high binding affinity and transport capacity, in order to increase the absorption of nitrate [110]. AtCIPK8 may participate in response to high concentrations of nitrate by perceiving and activating the low affinity nitrate reaction [111]. Experiments on the *cipk8* mutant showed that AtCIPK8 participates in the long-term process of nitrate-regulating root growth, and the response to primary nitrate produced a positive effect [111].

### 3.4. Phosphorus

Phosphorus is an essential nutrient for plant growth and development that accounts for about 0.2% of a plant’s dry weight [112]. It is also a constituent of nucleic acids and membrane phospholipids. High efficiency P nutrition in plants is related to many factors, such as root morphology and root exudates [113]. Plants can absorb Pi from the soil as an inorganic orthophosphate ion, but the availability is strictly limited by reactions of inorganic and organic phosphates with soil constituents [114].

The CBL−CIPK system is also involved during the response to low Pi in *Brassica napus* [115]. Yeast two-hybrid analysis revealed that BnCIPK6 is able to interact with *Arabidopsis* CBL1, CBL2, CBL3 and CBL9. BnCBL1 and BnCIPK6 were upregulated in Pi deficiency treatment and both proteins interacted with each other in yeast two-hybrid screens and a split-yellow fluorescent protein (YFP) system. This means that BnCBL1 and BnCIPK6 regulate the processes involved in the plant’s response to Pi deficiency [115]. Overexpression of either BnCBL1 or BnCIPK6 can enhance growth and biomass production under low Pi stress in *Arabidopsis* [115].

### 3.5. Magnesium

Although plants rely on a sufficient supply of Mg^2+^ for normal growth and development, excessive Mg^2+^ accumulation often causes toxicity to plant cells [116]. Two CBL proteins, CBL2 and CBL3, act as key regulators for plant growth under high-Mg conditions [117]. The *cbl2 cbl3* double-mutant plants retained a lower Mg content than wild-type plants under either normal or high-Mg conditions, suggesting that CBL2 and CBL3 may be required for vacuolar Mg^2+^ sequestration [117]. Four CIPKs; CIPK3, CIPK9, CIPK23 and CIPK26, act as functionally overlapping components downstream of CBL2/3 in the signaling pathway that facilitates Mg2^+^ homeostasis [117]. This Mg^2+^ partitioning process in the vacuole, controlled by the CBL−CIPK pathway, may represent a general mechanism underlying detoxification [118].

### 3.6. Calcium

Calcium acts as a critical messenger in the adaptation and developmental processes of plants. Generally, Ca^2+^ transmits the stress signal through a downstream pathway by binding to protein sensors called CBL, which interact with CIPKs, a specific group of protein kinases [75]. In plants, the protein CBL family represents a unique group of calcium sensors and helps to decode calcium transients by specifically interacting with, and regulating, a family of protein kinases (CIPKs). The unique feature of the *cbl10* mutant, despite being more sensitive to salt, is that it accumulates significantly less salt than the wild-type [119]. Different CBLs and their target kinases have been shown to function in stress responses. CBL4 (SOS3), and its interacting kinase CIPK24 (SOS2), together with SOS1, constitute a pathway that may function in Na+ exclusion from the cytoplasm [2]. 

Ca^2+^-dependent pathways always play critical roles in salt stress responses [120]. Ca^2+^ is the second most important messenger in terms of abiotic stress responses, such as salinity tolerance [121]. In the case of Na^+^ ion exclusion from the cytoplasm, Ca^2+^ sensor protein CBL4 (SOS3) interacts with the protein kinase CIPK24 (SOS2) and another Na^+^/H^+^ exchanger pathway (SOS1) at the plasma membrane [122]. The Ca^2+^-stress signaling system is complex; the Ca^2+^ signaling process is activated with the presence of a Ca^2+^ sensor and their target proteins.

## 4. Discussion

Plants are exposed to various adverse stress conditions during their growth and development processes. The stress sensor identification remains a vital part of abiotic stress research. Through mutant analysis and the CRISPR/CAS9 approach, an increasing number of genes that are relative to stress sensors will be studied deeply.

The discovery of the CBL and CIPK network represents an example of a significantly diverged Ca^2+^-decoding model for stress sensing that is distinct in plants. It is a key signaling system in various stress signaling pathways in plants [123]. Different CBL proteins have been reported to interact with different CIPK proteins, and the types of this interaction determine the network outcome [122]. To date, CBL and CIPK members have been comprehensively analyzed in many species (Table 2). In rice, there are more than 30 *OsCIPKs*, most of which respond to at least one stress factor among salt, drought and other abiotic stresses. *OsCIPK3*, *OsCIPK12* and *OsCIPK15* function as positive regulators of cold, drought and salt stress tolerance, respectively [124]. *OsCIPK3* was shown to negatively regulate salt stress tolerance in rice [125]. During the seed germination and seedling stage, *OsCIPK31*/*OsCK1* was associated with abiotic stress responses, which can lead to the differential expression of various stress-responsive genes [126]. *OsCIPK14* and *OsCIPK15* play an important role in the defense signaling pathway triggered by microbes in cultured rice cells [127].

Many genes for putative CIPK proteins were found in the *Arabidopsis* genome. In previous studies, the exchange of sodium (Na^+^), potassium (K^+^) and nitrate (NO^3−^) ion transportation across the plasma membrane and tonoplast in *Arabidopsis* was demonstrated to be regulated by the CBL−CIPK pathway [133]. cDNA cloning and sequencing have been confirmed in at least 25 *CIPK* genes. The interaction of CIPK24/SOS2 and CBL4/SOS3 was identified by a genetic screen that highlighted a role in salt tolerance in *Arabidopsis* [136]. In this connection, studies have established that CBL4/SOS3−CIPK24/SOS2 directly regulates the downstream component SOS1, a putative Na^+^/H^+^ antiporter that interacts with the Na^+^ ion detoxification process [137], as well as low-K-tolerant mutants

CIPK23, a critical K-nutrition determinant in *Arabidopsis*, was identified in a forward genetic screen [78]. CBL10, in a salt tolerant pathway, was mainly expressed and functioned in the shoots and leaves, unlike CBL4, another salt tolerant pathway, that only worked in the roots. The CBL4 (SOS) pathway interacted with CIPK24 (SOS2) protein kinase to export salt from the plasma membrane [119]. These studies support the idea that CBL−CIPK networks play important roles in abiotic stress sensing and regulating ionic balances.

During evolution, plants adopt different morphological and physiological changes in response to different abiotic stresses. Research has revealed that CBL−CIPK networks are integral in enabling plants to respond to abiotic stress and coordinate essential defense mechanisms [133]. By better understanding the CBL−CIPKs stress-sensing pathway, the ionic stress response mechanism of plants should be easily revealed. Previously, most of the research in CBL and CIPK focused on identifying the interactions between CBLs and CIPKs, the location of their interaction and the phenotypic analysis of CBL or CIPK mutants exposed to different abiotic stresses [80]. Further experiments have extended the analysis of CBL−CIPK interactions to the entire family of CBLs and a large fraction of the CIPK family in an effort to determine functional pairs of CBLs and CIPKs [120]. However, determining the downstream targets is essential to gaining a complete understanding of the CIPK and CBL signaling network.

## Figures and Tables

**Table 1:** Different transcription factors (TFs) and loci in different plant species.

Taxonomic Group	Latin Name	Species	TFs	Loci	Families
Monocots	*Oryza sativa* subsp. *japonica*	*Rice*	2408	1862	56
	*Oryza sativa* subsp*. indica*		1891	1891	56
	*Sorghum bicolor*	*Sorghum*	2654	1859	56
	*Triticum aestivum*	*Wheat*	3606	3606	56
	*Zea mays*	*Maize*	3308	2289	56
Eudicots	*Arabidopsis thaliana*	*Arabidopsis*	2296	1717	58
	*Nicotiana tabacum*	*Tobacco*	5176	3625	57
	*Solanum lycopersicum*	*Tomato*	1845	1845	58
	*Solanum tuberosum*	*Potato*	2405	1736	56

**Table 2:** Calcineurin B-like proteins (CBL) and CBL-interacting protein kinase (CIPK) members in different species.

Species	CBLs	CIPKs	Reference
*Arabidopsis thaliana*	10	26	[120,128,129]
*O. sativa*	10	31	[128]
*Triticum aestivum* L.	7	20	[130]
*Z. mays*	8	43	[131]
*Populus trichocarpa*	10	27	[132,133]
*B. napus* L.	7	23	[129]
*S. melongena* L.	5	15	[134]
*Malus domestica*	-	34	[135]
